# Oxidizability of Oils Recovered from Olive Seeds by Isothermal Calorimetry

**DOI:** 10.3390/foods11071016

**Published:** 2022-03-30

**Authors:** Ornella Kongi Mosibo, Siwawoot Laopeng, Giovanna Ferrentino, Matteo Scampicchio

**Affiliations:** Faculty of Science and Technology, Free University of Bozen-Bolzano, Piazza Università, 39100 Bolzano, Italy; ornella.mosibo@natec.unibz.it (O.K.M.); mr.siwawoot.laopeng@natec.unibz.it (S.L.); matteo.scampicchio@unibz.it (M.S.)

**Keywords:** oxidizability index, vegetable oils, isothermal calorimetry, extraction technologies

## Abstract

This work aims to apply isothermal calorimetry for the determination of the oxidative stability of bulk oils by deriving kinetic and thermodynamic parameters. The method consists of measuring the heat flow produced during the oxidation of the oils in the presence of oxygen. To this purpose, an oil was recovered from olive seeds, the solid waste derived from the transformation of olives, by using two different technologies: supercritical carbon dioxide and mechanical press. The oxidative stability of both extracted oils was then compared with commercial sunflower, soybean, corn, and rice oils. The kinetic and thermodynamic parameters, obtained from the analysis of isothermal calorimetry traces at 60 °C, allowed the calculation of the oxidizability index obtaining the following ranking: olive seeds by supercritical carbon dioxide (3.55 ± 0.4 × 10^−3^ (s/M)^0.5^) > sunflower (3.42 ± 0.8 × 10^−3^ (s/M)^0.5^) > olive seeds by mechanical press (3.07 ± 0.3 × 10^−3^ (s/M)^0.5^) > soybean (2.44 ± 0.6 × 10^−3^ (s/M)^0.5^) > corn (1.11 ± 0.4 × 10^−3^ (s/M)^0.5^) > rice oils (0.98 ± 0.4 × 10^−3^ (s/M)^0.5^). The results were then supported with the analysis of total phenolic content, antioxidant activity, fatty acid profile, and peroxide values. Overall, the findings of the present study support the use of isothermal calorimetry as a direct and non-invasive technique for determining the oxidizability of bulk oils.

## 1. Introduction

Olive stones or seeds represent a significant solid waste derived from the transformation of olives, especially in Europe. This waste originating from industrial plants needs particular attention, especially in Europe, because the EU produces roughly 67% of the world’s olive oil (Olive Oil European Commission). Olive seeds can be recovered intact along the olive oil production process, thanks to a destoning process where seeds are removed from the pulp. Olive seeds have peculiar properties that can be exploited by industries. They are, for instance, a good source of biomass for energy production, as shown in many scientific papers [1]. However, olive seeds can also be an interesting by-product for other reasons. Through their mechanical pressing, a natural and biological oil rich in nutrients and composed of high-value compounds can be extracted. Olive seed oil is rich in total polyunsaturated acids (PUFA). 1,2-Dilinoleoyl-3-oleoyl-glycerol is the major triacylglycerol, with the triacylglycerol species acylated with the linoleoyl chain being the most abundant in seed oil [2]. On the other hand, it is poor in total aliphatic long-chain and triterpene alcohols [3].

However, before assessing the beneficial aspects of the oil recovered from olive seeds, it is worth studying its stability to oxidation.

Lipid oxidation is likely the most important degradation reaction in bulk oils. The oxidation of PUFAs causes several undesirable effects on food quality and safety. The reaction is generally described by three successive periods, including initiation, propagation, and termination. The initiation period is induced by the formation of free radicals, which attack preferably the conjugated double bonds of polyunsaturated fatty acids in a chain mechanism, as by Ingold [4] and Denisov [5]. In food systems, lipid hydroperoxides (ROOH) are generally the primary products of lipid peroxidation and a source of peroxyl radicals. The accumulation of hydroperoxides requires a minimum partial pressure of oxygen (100 mm Hg. The accumulation characterizes the initial period of the propagation phase, which involves the attack of peroxyl radicals (ROO*) to RH and the formation of ROOH and alkyl radicals (R*). The analysis of lipid oxidation provides several kinetic and thermodynamic parameters which could help the understanding of oxidative stabilities.

Different techniques have been used to study the oxidizability of oils. The degradation of the lipid substrate is generally assessed by following the fatty acid breakdown or the evolution of volatile products. Due to the complex products formed during oxidation, these procedures entail further steps for the analysis of the various compounds obtained such as peroxide value (PV). This makes the process tedious and time-consuming and provides inaccurate conclusions on the oxidizability of oils. Furthermore, volatile analyses (e.g., chromatography) are more research-oriented than for quality control. They may also require trained and skilled people. To shorten the time issue, many accelerated methods have been developed to study the stability of oils such as the Schaal oven test and the active oxygen method (AOM). The Rancimat apparatus and the OSI (Oil Stability Index) apparatus are commonly used instruments applying these principles. Rancimat is widely used for vegetable oils and fish oils [6,7]. However, the method uses elevated temperatures and high oxygen supply which are different from the normal storage conditions. At higher temperatures, hydrogen peroxidation occurs at a faster rate, and secondary reactions such as polymerization also occur. For this reason, results reported by these methods may be arguable [6,8].

Hence, the aim of this work was to promote the ordinarily used methods of evaluating the oxidative stability of bulk lipid systems as well as to elucidate the capability of isothermal calorimetry to derive many kinetic parameters. To do this, four commercial edible oils (sunflower, soybean, corn, and rice) were oxidized over time. They were compared to the oil recovered from olive seeds using two different extraction methods: mechanical press and supercritical fluid extraction. The oil samples were also analyzed for their total phenolic content, antioxidant activity, fatty acid profile, and peroxide values.

## 2. Materials and Methods

### 2.1. Reagents

Four commercial oils (sunflower, soybean, corn, and rice) were purchased from a local supermarket. Folin–Ciocalteu’s reagent, gallic acid, 1,1-diphenyl-2 picrylhydrazyl (DPPH), Trolox (6-hydroxy-2,5,7,8-tetramethyl chroman-2-carboxylic acid), and fatty acid methyl esters (FAMEs) were purchased from Sigma Aldrich Chemical Co. (Milano, Italy). Solvents and all the other chemicals were of analytical grade.

### 2.2. Olive Seeds Preparation

Olive seeds were ground to obtain a homogeneous powder with the following particle size distribution: 50 ± 4% with a diameter higher than 1 mm and 50 ± 1% with a diameter between 1 mm and 500 μm (Retsch GmbH, Verder Scientific, Haan, Germany). The moisture content of the sample was equal to 8.2 ± 1.5% (Sartorious MA160, Torino, Italy) and the water activity to 0.73 ± 0.07 (AquaLab, Steroglass, Perugia, Italy).

### 2.3. Mechanical Press Extraction

The powdered seeds were then extracted with a mechanical press. The process was performed using a system with a pressing capacity of about 5 kg/day where the sample was slightly broken with rolling mills. The material was then sent to the mechanical press. During the extraction, the sample reached a temperature of 60 °C.

### 2.4. Supercritical Fluid Extraction

Supercritical fluid extraction (SFE) was carried out using a high-pressure pilot plant (Superfluidi s.r.l., Padova, Italy) equipped with 1 L volume extractor vessel and two gravimetric separators. The high-pressure vessel contained an extraction basket of 800 mL, closed with porous stainless steel mesh filters on both ends to enable carbon dioxide to pass the cylinder without transportation of solids to the exterior. The temperatures of the extractor and the two separators were automatically regulated through the recirculation of thermostated water from two individually regulated water baths. The CO_2_ was pressurized by a high-pressure diaphragm pump (Lewa LDC—M—9XXV1, Milano, Italy) precooled to 4 °C using a cooling heat exchanger to deliver liquid CO_2_ from a storage cylinder to the extraction vessel. The system was operated by passing supercritical state CO_2_ through a fixed bed of sample particles, precipitating liquid extract in the separators and finally releasing CO_2_ to the ambient surroundings. About 80 ± 1 g of ground olive seeds were filled inside the extractor. Optimal process conditions (30 MPa, 40 °C, 130 min, and CO_2_ flow rate equal to 4 L/h) were chosen according to previous studies [9,10].

### 2.5. Fatty Acid Composition

Fatty acids were determined by gas chromatography with flame ionization detector (GC-FID) using a chromatograph (Thermo Scientific TRACE 1300, Milano, Italy) and a fused silica capillary column (TG-POLAR, Thermo Scientific, 60 m, 0.25 mm and 0.2 μm). Hydrogen was used as carrier gas at a flow rate of 3 mL/min. To perform the analysis, 50 mg of oil was mixed with 4 mL of hexane, to which 150 μL of KOH solution (2 M in methanol) was added. The mixture was shaken for 10 min at ambient conditions and the upper layer was transferred to the GC vials. A volume of 1 μL was injected into the column through a split injector (1 of 10 μL) heated at 260 °C. Once injected, the sample passed through a column with the following temperature gradient: 50 °C for 1 min, 175 °C at a rate of 5 °C/min and 250 °C at 1 °C/min. The fatty acids methyl esters (FAME) of the samples were identified by comparing their retention times with standards (SUPELCO FAMEs Mix GLC-30; Sigma Aldrich, Milano, Italy). The determination was performed by measuring the area, expressed as percentage of normalized areas of the total fatty acids.

### 2.6. Peroxide Value Measurement

The peroxide value (PV) of the oils was measured based on the ferric thiocyanate method (IDF standard 74A:1991 2005). Briefly, an oil sample (5 μg) was transferred into glass tubes containing 10 mL chloroform–methanol (7:3, *v*/*v*). The mixture was shaken with a vortex mixer for 2–4 s. Later, ammonium thiocyanate solution (50 μL, 30% *w*/*v*) was added, and the solution was vortexed once again for 2–4 s. Finally, an aliquot of iron (II) solution (50 μL) was added to the glass tubes. For the iron (II) chloride stock solution, two solutions were made. For the first solution, 0.34 g of barium chloride was dissolved in 50 mL deionized water. For the second, 0.5 g of FeSO_4_·7H_2_O was dissolved in 50 mL deionized water. Finally, the barium chloride solution was added slowly, under constant stirring, to the iron (II) sulfate solution and to conclude, 2 mL of hydrochloric acid (10 N) was added to the mixture. The resulting solution formed a barium sulfate precipitate. To get a clear iron (II) solution, a filtration step was performed using a cellulose filter (Whatman, 15 cm diameter, GE Healthcare, Chalfont St Giles, UK).

The resulting reaction mixtures were stored in the dark at room temperature. The absorbance was read at 500 nm by a UV-VIS spectrophotometer (Cary 100, Agilent Technologies, Milano, Italy). Blank solutions were made up of all the reagents except the oil sample. A calibration curve was built using standard solutions of Fe(III) (1–40 μg/mL). Results were expressed in milliequivalents of peroxide per kilogram of oil [11].

### 2.7. Preparation of Extracts

Oil samples were extracted with methanol to recover phenolic compounds following the procedure of Siger et al. [12]. Oil samples (5 g) were dissolved in 50 mL of methanol and sonicated for 5 min, then centrifuged (Thermo Fisher, Milano, Italy) at 7000 rpm (4624× *g*) for 10 min and the supernatant transferred into amber bottles. The extraction was repeated three times. The methanolic extracts were combined, the solvent was evaporated by nitrogen flow, and the final extract was redissolved in methanol (5 mL final volume).

#### 2.7.1. Total Phenolic Content

The total phenolic content of oil methanolic extracts was measured by the Folin–Ciocalteu method [13]. Standard solutions of gallic acid (0.025–0.4 mg/mL) were prepared for the calibration curve. In a cuvette, 1.2 mL of distilled water was mixed with 40 μL of extract solution (or gallic acid solution). In total, 300 μL of carbonate solution and 100 μL of Folin–Ciocalteu reagent was added simultaneously. The mixture was kept in the dark for 2 h and the absorbance was recorded at 765 nm using a spectrophotometer (Cary 100 Series UV-Vis Spectrophotometer, Agilent Technologies, Milano, Italy). The results were expressed as mg of gallic acid equivalent (GAE)/g of oil.

#### 2.7.2. DPPH Radical Scavenging Activity

The antioxidant activity of oil methanolic extracts was estimated using 1,1-diphenyl-2-picrylhydrazil (DPPH) as described by [14] with small modifications [15]. Trolox (6-hydroxy-2, 5, 7, 8-tetramethylchroman-2-carboxylic acid) solutions (0–0.2 mg/mL) were prepared for the calibration curve. A total of 1.9 mL of DPPH (100 mg DPPH in 250 mL ethanol) solution was mixed with 100 μL of the samples (or Trolox solution) into a cuvette. The samples were kept in dark for 30 min at room temperature. The absorbance was measured at 515 nm with a spectrophotometer (Cary 100 Series UV-Vis Spectrophotometer, Agilent Technologies, Milano, Italy). Measurements were performed in triplicate and the results were expressed as mg of Trolox equivalent/g of oil.

### 2.8. Autoxidation Experiments

Autoxidation experiments of oils were performed using a microcalorimeter (Thermal Activity Monitor, Model 421 TAM III, TA Instruments, Milano, Italy) equipped with 24 channels operating in isothermal mode. Glass ampoules (4.0 × 10^−3^ L) were accurately filled with 100 mg of each oil. A duplicate of each experiment was also carried out. The ampoules were maintained at a constant temperature (60 °C with an absolute accuracy of ±0.0005 °C) in the thermostat. Initially, an equilibration step was carried out for 15 min. During this step, the ampoule was held at mid height inside the channel to allow the equilibrium between the temperature of the sample and the thermostat. Later, the ampoule was fully lowered into the measuring position for the whole duration of the experiment. The heat flow was then recorded over time at a 10 s interval.

### 2.9. Statistical Analysis

Results are expressed with mean and standard deviations and were analyzed by XLSTAT (Version 2016.02.28014, New York, NY, USA) by applying ANOVA analysis and detecting the significant differences (*p* < 0.05) by Tukey test. Graphics were performed with R [16].

## 3. Results and Discussion

### 3.1. Isothermal Calorimetry Properties of Olive Seed Oils

**Figure 1:**
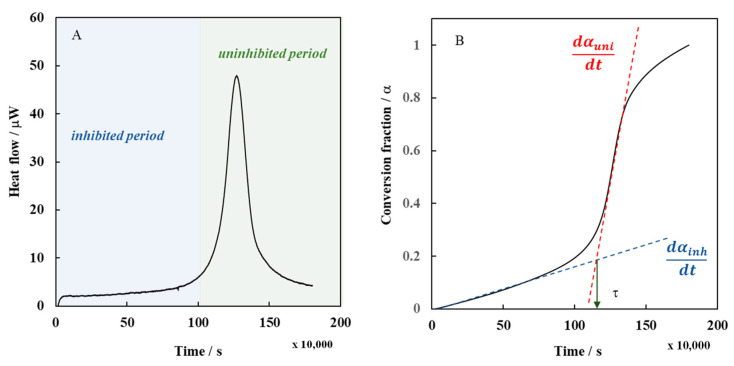
Representative calorimetric trace of olive seed oils obtained by mechanical press. (**A**) Heat flow data; (**B**) Conversion fraction derived from the integration of heat flow data. Tangent lines to the conversion fraction correspond to the $\alpha_{inh}$ and $\alpha_{uni}$, the rate of lipid oxidation during the inhibition and uninhibited periods, respectively. τ is the induction period.

The oxidizability of bulk olive seed oil was studied by isothermal calorimetry. Figure 1A shows a representative transient heat-flow signal recorded for an olive seed oil extracted by mechanical pressing and maintained in isothermal conditions in a closed ampoule at 60 °C. The transient signal shows a relatively long initiation period characterized by a nearly constant heat flow value, followed by a sudden increase, which reached a maximum point, corresponding to the maximum heat flow value. Finally, the rate of heat generation rapidly decays, indicating the achievement of a rate-limiting condition, i.e., when the oxygen in the ampoule was completely depleted. The fact that the heat flow signal did not completely return to a zero value implies that other reactions, overall exothermic, may occur even in the later periods of oxidation when the partial pressure of oxygen was negligible. In Figure 1B, the previous calorimetric trace was converted into conversion fraction. The conversion fraction (*α*) or extent of the reaction is a value that changes from 0 to 1 during the conversion of reactants to products. It was calculated by dividing the accumulated heat (i.e., cumulative integration of the heat flow rate) by the overall heat (i.e., the integrated heat flow from the calorimetric trace), as defined in Equation (1):

(1) $$\alpha =\frac{q\left(t\right)}{Q_{tot}}$$

It should be noted that at the beginning of the experiment, the oxidation of olive seeds oil did not start immediately. This was because the oils contained compounds (phenols, tocopherols, carotenoids and other minor components) with antioxidant activity acting as stabilizers. Until they were present, the conversion fraction remained zero. This corresponded to the rate of lipid oxidation during the inhibited period (*dα*/*dt*)*_inh_* and revealed how fast the antioxidants contained in the oil can inhibit the radical chain process. Only when the action of these compounds was depleted, the uninhibited autoxidation of the oil could begin (*dα*/*dt*)*_uni_*. From that point on, the initial heat flow value and the conversion fraction increased linearly and steadily.

### 3.2. Kinetic and Thermodynamic Properties of Olive Seed Oils

From the calorimetric traces recorded from olive seed oil samples, and from the chemical properties of the bulk oil (Table 1 and Table 2), we derived both kinetic and thermodynamic properties reported in Table 3. The following section aims to exemplify how such properties (induction time, enthalpy of reaction, rate of radical initiation, rate of inhibited and uninhibited periods) were derived.

#### 3.2.1. Induction Time

The induction time of this calorimetric trace is the simplest parameter to derive, and likely the most important, to characterize the oxidative stability. Similarly, to other instruments devoted to monitoring lipid oxidation processes, such as Rancimat [17] or Oxipress [18], the induction time was obtained from the length of time between the zero point on the abscissa and the point of intersection of the two tangent lines drawn, respectively, to the early stages of the inhibited and uninhibited periods. The resulting induction time of olive seeds oils obtained by mechanical press was 1.13 ± 0.5 × 10^6^ s. This is the time when all the antioxidants present in the bulk oil were consumed. Afterwards, the oxidation of the oils could proceed freely at its maximum rate. It should be highlighted that, differently from the Rancimat or Oxipress methods, the proposed approach based on isothermal calorimetry employed conditions of temperature and pressure milder and closer to those typically found during the normal storage of foods. This makes isothermal calorimetry more advantageous and the results less prone to artifacts.

#### 3.2.2. Overall Heat of the Reaction

The overall heat generated during the reaction between olive seeds oil and oxygen was *Q_tot_* = 14.1 ± 0.9 J (*n* = 6). This value reflects all the many reactions, overall exothermic, that occurred simultaneously in the bulk oil, such as the formation of hydroperoxides, volatile compounds, and peroxyl radicals. However, at the end of the calorimetry experiment, the partial pressure of oxygen inside the ampoule was negligible (by oximetry). Accordingly, given the amount of oxygen in the headspace of the glass ampoule, which was equal to 3.0 × 10^−5^ mol of oxygen (*n* = P·V_head space_/R·T, with P = 0.21 atm, V_head space_ = 3.9 × 10^−3^ dm^3^, T = 333 K, and R = 0.082 dm^3^ atm/K mol), it was possible to express overall heat of oil oxidation relative to the amount of oxygen consumed, leading to a specific heat of 470.1 kJ/mol.

#### 3.2.3. Rate of Initiation

The rate of chain initiation (*R_i_*) reflects how fast radicals are produced. This can be estimated based on the following Equation (2):

(2) $$R_{i}=\frac{n\times \left[ArOH\right]{\;}_{}}{\tau }$$

Which expressed the ratio between the total polyphenol equivalents extracted from the oils (*n* × [*ArOH*]) and the induction time (*τ*). The total polyphenol content of olive seed oils was 1.00 ± 0.2 g GAE/kg of oil (Table 3). Assuming an apparent stoichiometry factor, n of 2.0 (as suggested by many previous works for the analysis of phenolic acids and tocopherols [19,20]), the rate of chain initiation of olive seed oils could be estimated as 8.7 ± 0.4 × 10^−9^ M/s.

#### 3.2.4. Rate of the Inhibited Period

The rate of lipid oxidation during the inhibited period (*R_inh_*) reveals how fast the antioxidants contained in the oils can inhibit the radical chain process. This information can be directly obtained by isothermal calorimetry from the slope of the initial linear tract of the time conversion curve (e.g., the tangent line drawn at the beginning of the oxidation period of olive seeds oil) (Figure 1B). The slope was equal to 2.94 ± 0.08 × 10^−7^ s^−1^, and corresponded to the apparent conversion rate (*dα*/*dt*) of the inhibited autoxidation of olive seeds oil:

(3) $$\frac{R_{inh}}{{\left[O_{2}\right]}_{0}}={\left(\frac{d\alpha }{dt}\right)}_{inh}$$

The multiplication of the apparent conversion rate by the moles of oxygen initially present in the headspace of the ampoule (*n* = P_O2_∙V_head space_/R∙T = 3.0 × 10^−5^ mol) provides the initial rate of oxygen consumption during the inhibited autoxidation of the oil, which was equal to *R_inh_* = 8.82 ± 0.26 × 10^−12^ mol∙s^−1^. Such multiplication was assumed valid at least during the initial period of the experiment, where oxygen concentration was not limiting the oil oxidation. Finally, the division of such rate by the volume of the sample (V_sample_ = 1.11 ± 0.1 × 10^−4^ L) allowed to express *R_inh_* as 7.94 ± 0.35 × 10^−8^ M s^−1^.

#### 3.2.5. Rate of the Uninhibited Period

A further kinetic parameter that characterizes the oxidation process of bulk oils is the rate of the uninhibited period (*R_uni_*). The conversion rate of the uninhibited oil autoxidation (e.g., when all the antioxidants are consumed) was calculated from the slope of the conversion fraction during time in the second linear tract of the curve (e.g., the tangent line drawn after the induction time of the oxidation period). The slope resulted equal to 2.99 ± 0.2 × 10^−6^ s^−1^, and corresponded to the apparent conversion rate (*dα*/*dt*) of the uninhibited autoxidation of olive seeds oil extracted by mechanical press (Equation (4)):

(4) $$\frac{R_{uni}}{{\left[ROOH\right]}_{0}}={\left(\frac{d\alpha }{dt}\right)}_{uni}$$

The multiplication of the apparent conversion rate by the moles of peroxydes [*ROOH*]_0_ formed after the consumption of all the antioxidant present in the oil ([*ROOH*]_0_ = 2.90 × 10^−3^ M, obtained by converting the peroxide value from milli eq./kg in mmol/L and multiplying by the density of the oil) allowed to express the rate of the uninhibited autoxidation of olive seeds oil, which was equal to *R_uni_* = 0.86 ± 0.1 × 10^−8^ M∙s^−1^.

#### 3.2.6. Oxidative Stability Index

The oxidizability index of the oils was proposed by [21,22,23,24] and based on the following Equation (5):

(5) $$O.I.=\frac{\;R_{uni}}{\left[RH\right]\;\cdot \sqrt{R_{i}}}$$

This is expressed as the ratio between the rate of lipid oxidation during the uninhibited period, *R_uni_*, and the product between the rate of initiation *R_i_* and the initial concentration of oxidizable substrates. For olive seed oils, the initial concentration of oxidizable substrates was equal to [*RH*] = 0.03 M. Accordingly, the resulting oxidizability index was equal to 3.07 ± 0.3 × 10^−3^ (s/M)^0.5^.

### 3.3. Application to Bulk Edible Oils

**Figure 2:**
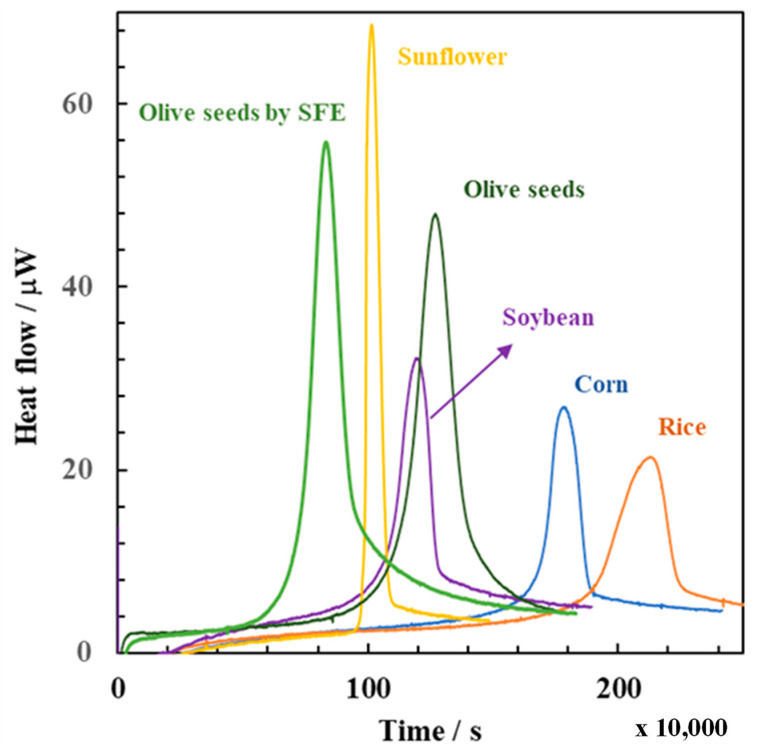
Isothermal calorimetry traces of bulk oils at 60 °C.

The approach previously described was next applied to retrieve detailed information on the oxidizability of different bulk edible oils. Figure 2 shows the typical calorimetric traces recorded for the autoxidation of sunflower, soybean, corn, rice, and olive seed oils, the latter either extracted by mechanical press or by supercritical carbon dioxide. From such traces, Table 3 reports the corresponding conversion rates of the uninhibited (*dα*/*dt*)*_uni_* and inhibited period (*dα*/*dt*)*_inh_* of the oils autoxidation, the overall heats (Q_tot_) and the induction times (*τ*). Since it is known that the rate of autoxidation depends on several factors, such as the amount of unsaturated fatty acids (mainly C18:2 and C18:13) and the content of strong antioxidants that inhibit radical chain reactions, accordingly, Table 1 reports the results of the total phenolic compounds (by Folin Ciocalteu method), the total antioxidant capacity (by DPPH assay) and peroxide values. In addition, Table 2 reports the fatty acid profile of bulk oils, including the total amount of monounsaturated fatty acid (MUFA) and polyunsaturated fatty acids (PUFA).

Olive seed oil samples extracted by SFE showed the highest initial rate of radical productions (*R_i_*), followed by soybean > sunflower > olive seed by mechanical press > corn > rice oils. The *R_i_* value is an important parameter as it accounts for the amount of hydroperoxides initially present in the bulk oil, their apparent stoichiometry number (i.e., the average number of radicals species trapped by each antioxidant), and how fast free radicals are generated in the bulk oils. The latter can be estimated with the initial peroxide value, i.e., where a higher number of peroxides correspond to higher free radicals and faster degradation of antioxidants.

Table 4 also shows the rate of the inhibited period. This rate reflects the capacity of an oil to scavenge the free radicals that are formed. The lower is the rate of inhibition, the slower is the formation of peroxyl radicals; thus, this implies the highest oxidative stability. Accordingly, the most stable oils follow (from high to low): olive seeds by SFE > soybean > sunflower > olive seeds by mechanical press > corn > rice, which is similar to the ranking observed for the rate of initiation, although obtained with independent approaches.

Finally, Table 4 also reports the results of the rate of propagation phase of lipid peroxidation and the oxidizability index. Each piece of information is also important because they describe the oxidizability behavior of the oil. In general, the propagation phase occurs when free radicals formed in the bulk oil can freely react with the lipid substrates without the presence of antioxidants. During the propagation period, the rate of lipid oxidation is the highest. Indeed, based on isothermal calorimetry data, the oil that yielded the highest *R_uni_* values was sunflower oil with 2.17 ± 0.1 × 10^−8^ M^−1^s^−1^ followed by soybean, olive seeds by SFE, and olive seeds by mechanical press with *R_uni_* values of 1.64 ± 0.1 × 10^−8^ M^−1^s^−1^, 1.61 ± 0.3 × 10^−8^ M^−1^s^−1^ and 0.86 ± 0.1 × 10^−8^ M^−1^s^−1^, respectively. The last positions were occupied by corn and rice oils with *R_uni_* values of 0.66 ± 0.4 × 10^−8^ M^−1^s^−1^ and 0.51 ± 0.1 × 10^−8^ M^−1^s^−1^.

Finally, the trend of the oxidizability index followed a ranking very similar to the *R_uni_* values with the subsequent order: olive seeds by SFE (3.55 ± 0.4 × 10^−3^ (s/M)^0.5^) > sunflower (3.42 ± 0.8 × 10^−3^ (s/M)^0.5^) > olive seeds by mechanical press (3.07 ± 0.3 × 10^−3^ (s/M)^0.5^) > soybean (2.44 ± 0.6 × 10^−3^ (s/M)^0.5^) > corn (1.11 ± 0.4 × 10^−3^ (s/M)^0.5^) > rice oils (0.98 ± 0.4 × 10^−3^ (s/M)^0.5^).

### 3.4. Correlation between Isothermal Calorimetry Data and Chemical Properties of Bulk Oils

Based on the induction times (Table 3), the oils obtained by mechanical pressing from olive seeds (*Olea europaea* L.) were relatively stable, with an induction time observed at about 1.13 ± 0.5 × 10^6^ s at 60 °C. What is striking here is that the extraction of the same olive seeds by supercritical carbon dioxide resulted in a significantly lower induction time ((0.32 ± 0.1) × 10^6^ s), and, thus, in a much lower oxidative stability. The main chemical characteristic that differentiates the oxidative stability of the two oils (obtained from the same seeds) is the total polyphenol content. Table 1 shows that the oils obtained by supercritical fluids show a significant decrease (about 70%) of total polyphenol content and antioxidant activity. Such reduction is justified if we consider that supercritical carbon dioxide is a nonpolar solvent. Accordingly, without the addition of a co-solvent such as ethanol or methanol, supercritical carbon dioxide is scarcely able to extract those hydrophilic phenols, which are responsible for most of the antioxidant properties of olives [25,26]. However, phenols alone cannot explain the resulting oxidative stability of all the oils considered in this study.

For instance, sunflower oil appeared as the most unstable one among the others (see Figure 2). Conversely to the olive seed oils, the high rate of oxidation exhibited by sunflower oil can be explained not only because of the low content of total phenols (0.98 ± 0.05 GAE/kg) and the low antioxidant activity (0.48 ± 0.03 g TE/kg) (Table 1) but also because of the high content of PUFA (12.4 ± 2.1 g/L). On one side, the low total phenolic content in commercial sunflower is attributed to the refining processes (i.e., degumming, neutralization, bleaching and deodorization), which is needed to remove the undesirable flavors and odors of the raw oil, and which is responsible to degrade more than 40% and 60%, respectively, of the total phenolic and flavonoid contents initially present in the seeds [27]. On the other side, the high rate of oxidation of sunflower oils can be explained by the presence of large quantities of polyunsaturated fatty acids, mainly α-linolenic. Not surprisingly, recent trends in the processing of edible sunflower oils tend to prefer those varieties that are naturally rich in oleic acid, which show better resistance to oxidative degradation even during frying [28].

Soybean oil was more stable than sunflower oil (Figure 2). This was unexpected based on fatty acid analysis (Table 2). In comparison with sunflower oil, soybean oil showed not only an overall higher content of PUFA (11.0 ± 1.5 g/L) but especially one of the highest contents of linolenic acid (C18:3) among the oils analyzed in this work. Similar results were reported by differential scanning calorimetry (DSC) measurements, under non-isothermal conditions, concluding that the oxidative stability of soybean oil was higher than sunflower oil because sunflower oil contained a higher quantity of linoleic acids [29]. However, this conclusion is not clear if we consider the higher content of linolenic acid, which is a conjugated fatty acid having a propagation rate constant that is four times higher than that of linoleic acid and more than 200 times higher than that of oleic acid at 303 K [19]. The reason for such higher stability must be explained by the presence of strong antioxidants, which can effectively intercept peroxyl radicals by transferring the H-atom at a rate faster than that of chain propagation. This conclusion was supported by other authors that analyzed the presence in refined soybean oils of relatively high levels of tocols, among which tocopherols were by far most relevant [30]. It is worth noticing that α-tocopherol, one of the most representative antioxidants found in soybean oils, has an inhibition rate constant of more than 3.0 × 10^6^ M^−1^s^−1^ at 37 °C, which is about 50.000 times faster than the propagation rate constant for the peroxidation of linoleate (*k_p_* = 62 M^−1^s^−1^) [31]. This explains the resulting stability of the oil.

A similar conclusion can be derived for the oxidative stability exhibited by corn oil (the oil extracted from the germ of maize), which is the second most stable oil among the edible oils analyzed in this work. The high oxidative stability reflects the high levels of total phenols (1.54 ± 0.1 g GAE/kg (Table 1), whose values are consistent with those reported previously [12,32]. The high content of total phenols, together with the high levels of tocopherols [33] stabilizes the high content of PUFA (12.5 ± 2.5 g/L) and explains its superior oxidative stability. Also, previous results obtained by the Rancimat test and the thermostat test, showed that corn oil, together with rice oil, showed the slowest changes in peroxide values and the longest storage stability among several edible oils [33].

Finally, rice oil was the most stable one. The superior stability of rice bran oil observed from Figure 2 can be explained, similarly to the other oils, by a combination of fatty acid composition (high levels of oleic acid, lower levels of linoleic acid, and α-linolenic acid, see Table 2) together with high total antioxidant capacity (Table 1). These conclusions agree well with previous storage stability studies, based on peroxide values, and show that the most stable oils were rice > soybean > sunflower oils [34].

## 4. Conclusions

The present study is an attempt to promote the use of isothermal calorimetry to characterize the oxidizability of bulk lipid systems. The kinetic and thermodynamic parameters that can be easily obtained from isothermal calorimetry data provide quantitative and objective criteria to better comprehend and predict the oxidizability of bulk lipid systems. Conversely, studies based on the chemical compositions (i.e., fatty acid profile and peroxide values), phenolic content, and antioxidant capacity cannot predict, if taken individually, the complex nature of bulk oils. Overall, the methodological approach shown in this work provides advice for researchers in their future studies and promotes the use of isothermal calorimetry able to provide comprehensive information, generally achievable only by performing multiple testing methods, such as total phenolic content, DPPH assays, fatty acid profile, and peroxide values. Our findings support the use of isothermal calorimetry as a universal approach for determining the oxidizability of bulk oils.

## Figures and Tables

**Table 1:** Values of the total polyphenol content obtained by Folin–Ciocalteu, antioxidant activity obtained from DPPH and peroxide values of bulk oils. Results are expressed as mean values ± standard deviations.

Oil	Folin–Ciocalteu	DPPH	Peroxide Value
	g GAE/kg	g TE/kg	Millieq. Peroxide/kg
Olive seed	1.00 ± 0.2	0.60 ± 0.03	6.44 ± 0.3
Olive seed from SFE	0.36 ± 0.2	0.20 ± 0.01	6.88 ± 1.9
Sunflower	0.98 ± 0.1	0.48 ± 0.03	5.19 ± 2.8
Soybean	1.21 ± 0.5	0.65 ± 0.04	10.91 ± 1.5
Corn	1.54 ± 0.1	0.71 ± 0.05	6.67 ± 0.9
Rice	1.26 ± 0.1	1.00 ± 0.06	5.62 ± 1.3

**Table 2:** Fatty acid composition of oils. Results are expressed as mean values ± standard deviation (*n* = 3).

Oil	MUFA	PUFA	16:0	16:1	18:0	18:1	18:2	18:3
	g/L	g/L	(%)	(%)	(%)	(%)	(%)	(%)
Olive seed	6.8 ± 0.6	1.7 ± 0.5	8.6 ± 0.8	0.21 ± 0.02	2.75 ± 0.9	71 ± 2.4	17.1 ± 1.5	0.32 ± 0.01
Olive seed from SFE	8.37 ± 1.1	2.1 ± 0.4	8.1 ± 1.1	0.11 ± 0.01	2.75 ± 0.4	71 ± 2.3	16.8 ± 2.5	0.31 ± 0.03
Sunflower	5.9 ± 0.7	12.4 ± 2.1	6.1 ± 0.9	-	2.96 ± 0.11	28 ± 1.5	62.4 ± 2.7	-
Soybean	6.3 ± 1.5	11.0 ± 1.5	10.6 ± 1.5	-	4.23 ± 0.5	28 ± 1.9	50.7 ± 2.8	5.62 ± 0.06
Corn	6.7 ± 1.0	12.5 ± 2.5	11.7 ± 1.6	-	1.55 ± 0.3	29 ± 2.5	56.6 ± 3.5	0.82 ± 0.05
Rice	9.8 ± 1.8	7.2 ± 1.7	20.2 ± 1.4	0.18 ± 0.01	2.11 ± 0.3	44 ± 3.2	32.5 ± 2.3	1.05 ± 0.6

**Table 3:** Kinetic and thermodynamic data measured directly from isothermal calorimetry. *α_inh_* is the conversion fraction measured in the early tract of the inhibited period. *α_uni_* is the conversion fraction measured in the uninhibited period. *Q_tot_* is the overall area of the calorimetric trace, *τ* is the induction period. Results are expressed as mean values ± standard deviations.

Oil	${\left(\frac{d\alpha }{dt}\right)}_{inh}$	${\left(\frac{d\alpha }{dt}\right)}_{uni}$	*Q_tot_*	*τ*
	10^−7^ s^−1^	10^−6^ s^−1^	J	10^6^ s
Olive seed	2.94 ± 0.2	2.99 ± 0.6	15.1 ± 0.1	1.13 ± 0.5
Olive seed from SFE	4.18 ± 0.5	5.19 ± 1.2	14.1 ± 0.1	0.32 ± 0.1
Sunflower	3.02 ± 0.3	9.29 ± 1.7	6.1 ± 0.1	0.97 ± 0.3
Soybean	3.18 ± 0.2	2.71 ± 0.8	11.2 ± 0.1	1.11 ± 0.4
Corn	2.61 ± 1.1	2.21 ± 0.6	9.9 ± 0.1	1.70 ± 0.5
Rice	2.27 ± 0.4	2.01 ± 0.8	11.5 ± 0.1	1.91 ± 0.6

**Table 4:** Kinetic and thermodynamic properties of different bulk oils derived from the results in Table 3. Legend: *R_i_* is the rate of free radical formation, and it is calculated from 2[*ArOH*]/*τ*. *R_uni_* is the rate of lipid oxidation in the uninhibited period. *R_inh_* is the rate of lipid oxidation during the inhibited period, *R_uni_* is the rate of lipid oxidation during the uninhibited period. Finally, O.I. stands for the oxidizability index and it is measured as $R_{uni}/\sqrt{R_{i}}\cdot \left[RH\right]$.

Bulk Oil	*R_i_*	*R_inh_*	*R_uni_*	O.I.
	10^−9^ M/s	10^−8^ M/s	10^−8^ M/s	10^−3^ (s/M)^0.5^
Olive seed	8.7 ± 0.4	7.94 ± 0.15	0.86 ± 0.1	3.07 ± 0.3
Olive seed from SFE	15.3 ± 0.5	10.1 ± 0.25	1.61 ± 0.3	3.55 ± 0.4
Sunflower	9.5 ± 0.4	8.10 ± 0.45	2.17 ± 0.1	3.42 ± 0.8
Soybean	12.0 ± 0.5	8.42 ± 0.15	1.64 ± 0.1	2.44 ± 0.6
Corn	8.2 ± 0.4	7.06 ± 0.18	0.66 ± 0.4	1.11 ± 0.4
Rice	7.21 ± 0.5	6.08 ± 0.19	0.51 ± 0.1	0.98 ± 0.4

## Data Availability

The data presented in this study are available within this article.
